# Clinical and oncological outcomes in Chinese patients with renal cell carcinoma and venous tumor thrombus extension: single-center experience

**DOI:** 10.1186/s12957-015-0448-2

**Published:** 2015-02-04

**Authors:** Xiaonan Chen, Shijie Li, Zhenqun Xu, Kefeng Wang, Donghui Fu, Qiang Liu, Xia Wang, Bin Wu

**Affiliations:** Department of Urology, Shengjing Hospital of China Medical University, Shenyang, 110004 Liaoning People’s Republic of China

**Keywords:** Renal cell carcinoma, Venous tumor thrombus, Cancer-specific survival, Prognostic factors

## Abstract

**Background:**

To evaluate the clinical and oncological outcomes and to identify prognostic factors for survival in Chinese patients with renal cell carcinoma (RCC) and venous tumor thrombus (VTT).

**Methods:**

A total of 86 patients who underwent nephrectomy and tumor thrombectomy for RCC and venous tumor thrombus extension from 2003 to 2013 were included in this retrospective study. The records of these patients were reviewed. Kaplan-Meier analysis was used to determine cancer-specific survival (CSS). Prognostic factors for CSS were identified by univariate and multivariate analyses using the Cox proportional hazards regression mode.

**Results:**

All patients in this cohort received radical nephrectomy and tumor thrombectomy. Median follow-up period was 27.0 months (range 3–111). No patients died intraoperatively, and the complication rate was 36.0%. The 1-, 3-, and 5-year CSS rates for all patients were 93.0%, 70.9%, and 58.1%, respectively, and those for patients without distant metastasis at presentation were 95.3%, 82.6%, and 68.6%, respectively. Multivariate Cox regression analysis showed that lymph node invasion, distant metastasis at presentation, and invasion of the inferior vena cava (IVC) wall were the independent prognostic factors for CSS in all patients. For patients without distant metastasis, tumor grade, lymph node invasion, and perinephric fat invasion were significantly associated with CSS on multivariate analysis.

**Conclusions:**

Survival rates for patients with RCC and VTT were still poor. Our results indicated that lymph node invasion, distant metastasis at presentation, and invasion of the IVC wall were independent negative prognostic factors.

## Background

Renal cell carcinoma is a common solid tumor of the urinary system, and its incidence tends to rise year by year. It has been reported that approximately 4%–15% patients with renal cell carcinoma (RCC) were found to have venous tumor thrombus on diagnosis of the cancer [1]. Despite advances in targeted molecular therapy, radiotherapy, chemotherapy, and immunotherapy, surgical treatment is still the most important factor influencing the prognosis of patients with RCC [2]. With continuous improvements in surgical techniques and perioperative management, surgical morbidity and mortality rates are increasingly favorable in patients with RCC and venous tumor thrombus (VTT). On the other hand, the 5-year cancer-specific survival (CSS) is a mere 25%–65% in those patients with RCC and VTT following surgery [3-5].

Some prognostic factors have been reported in patients with RCC and VTT, mostly by retrospective studies based on small-sample sizes. However, the significance of these factors remains controversial. Some researchers have concluded that the level of venous tumor thrombus correlates negatively with prognosis [6], but others have held that the level of venous tumor thrombus is not an independent prognosis predictor [3,5]. Although there have been successful cases in which neoadjuvant-targeted molecular therapies have succeeded in lowering the level of tumor thrombus, making surgical treatment possible [7], the issue of whether such therapies are indicated for patients with Level III and IV tumor thrombus remains controversial [8]. Most studies on the prognosis (and its influencing factors) of patients with RCC and VTT have been conducted in Western countries, and there have been only a few studies conducted in Asian populations [3,9], in particular in Chinese populations [10]. In the present study, therefore, we retrospectively analyzed the clinicopathological data of patients with RCC and VTT who underwent surgical treatment at our center and assessed the effect and significance of each prognostic factor on these patients.

## Methods

From June 2003 to June 2013, 1,518 patients were diagnosed with RCC in our institution. Among these, a total of 91 patients underwent nephrectomy and tumor thrombectomy for RCC and VTT extension. Five patients were lost at follow-up, and 86 patients were included in this study. Those patients who were inoperable were not included in this study. The medical records were reviewed retrospectively for demographics, clinical symptoms at diagnosis, laboratory findings, performance status (PS) as defined by the Eastern Cooperative Oncology Group (ECOG] [11], and clinical tumor features including tumor size, tumor laterality, and maximal level of tumor thrombus. Histopathological features included tumor-node-metastasis (TNM) stage (according to the newly-revised 2009 AJCC TNM classification), histopathological subtypes, tumor grading (according to the 2012 International Society of Urological Pathology (ISUP) grading system) [12], invasion of renal pelvis, invasion of venous wall, and perinephric fat invasion. Informed consent for this study was obtained from each patient, and the study was approved by the Research Ethics Committee of our institution.

All patients preoperatively underwent routine blood tests, chest X-ray (computed tomography (CT) for selected patients), abdominal CT, ultrasound, and/or abdominal magnetic resonance imaging (MRI), and/or bone scanning. Postoperative follow-up included blood tests, chest and abdominal CT examination, and/or bone scanning, and/or brain MRI. The follow-up data was obtained during checkup visits and telephone interviews. The maximal level of tumor thrombus was classified according to the Mayo Clinical Classification [13]: level 0, thrombus extending to the renal vein; level I, tumor thrombus present either at the entry of the renal vein or within the inferior vena cava (IVC) <2 cm from the confluence of the renal vein and IVC; level II, tumor thrombus extending within the IVC >2 cm above the confluence of the renal vein and IVC but still remaining below the hepatic vein; level III, tumor thrombus involving the intrahepatic IVC; and level IV, tumor thrombus extending above the diaphragm or into the right atrium.

### Surgery

None of the patients with level IV tumor thrombus received surgical therapy in our institution. However, two patients demonstrated clinical thrombus regression from level IV to III after receiving targeted molecular therapies (2–3 cycles), and both of them then received surgical therapy. All patients enrolled in this study underwent radical nephrectomy and thrombectomy. The surgical approach was determined by the characteristics of the tumor and associated thrombus, as well as the experience of the surgeons. Twelve (14.0%) patients with complications of invasion of the IVC wall were detected, and all of these underwent partial resection of the IVC wall. Two of them needed reconstruction of the IVC with synthetic grafts due to the extensive IVC defect.

### Statistic analysis

The CSS was calculated using the Kaplan-Meier method, and the differences were determined by the log-rank test. The CSS was evaluated from the date of surgery to the last follow-up or death caused by RCC. Prognostic factors for CSS were identified by univariate and multivariate analyses using the Cox proportional hazards regression model, and hazard ratio (HR) with 95% confidence intervals were calculated. Differences were considered statistically significant at a *P* value of <0.05. All statistical analyses were performed using the SPSS software (version 22.0).

## Results

A total of 86 patients with RCC and VTT extension were included in this retrospective study. Baseline clinicopathological characteristics of the patients are summarized in Table 1. Male-to-female ratio was 2:1. A total of 47 cases (54.7%) of RCC occurred on the right side, and 39 cases (45.3%) occurred on the left. The mean age at diagnosis was 57.7 ± 11.3 years (range 15–79). The mean diameter of tumor was 8.2 ± 2.4 cm (range 3.2–14.0). The median follow-up period was 27.0 months (range 3–111). A total of 53 patients (61.6%) had a level 0 tumor thrombus, 16 (18.6%) had a level I thrombus, 11 (12.8%) had a level II thrombus, and 6 (7%) had a level III thrombus according to the Mayo Clinical Classification. Fifty patients (58.1%) were T3a stage, 17 (19.8%) were T3b, 10 (11.6%) were T3c, and 9 (10.5%) were T4 according to the newly revised 2009 AJCC TNM classification. The tumor was symptomatic in 47 cases (54.7%), whereas 39 cases (45.3%) were incidental findings. Thirty-one (36%) patients had perinephric fat invasion and ten (11.6%) had renal pelvis invasion. Pathology results showed that clear cell RCC was diagnosed in 67 (77.9%) patients and non-clear-cell subtypes in 19 (22.1%) patients.

**Table 1:** **Clinical and histopathological characteristics of patients**

**Variables**	**Total**	**Renal vein thrombus**	**IVC thrombus**	***P*** **value**
N (%)	86	53(61.6)	33(38.4)	
Age, year	57.7 ± 11.3	57.1 ± 11.5	58.7 ± 11.2	0.535
Gender (%)				
Male	58(67.4)	35(66)	23(69.7)	0.725
Female	28(32.6)	18(34)	10(30.3)	
Laterality (%)				
Left	39(45.3)	27(50.9)	12(36.4)	0.187
Right	47(54.7)	26(49.1)	21(63.6)	
Clinical symptom (%)				
Yes	47(54.7)	26(49.1)	21(63.6)	0.187
No	39(45.3)	27(50.9)	12(36.4)	
Tumor size, cm	8.2 ± 2.4	8.1 ± 2.2	8.3 ± 2.5	0.731
ECOG-PS	0.8 ± 0.8	0.6 ± 0.8	1.2 ± 0.7	0.04
Histological type (%)				
Clear cell	67(77.9)	43(81.1)	24(72.7)	0.361
Others	19(22.1)	10(18.9)	9(27.3)	
Tumor grade (%)				
G1	6(7.0)	6(11.3)	0(0)	
G2	38(44.2)	24(45.3)	14(42.4)	0.156
G3	33(38.4)	19(35.8)	14(42.4)	
G4	9(10.5)	4(7.5)	5(15.2)	
T stage (%)				
T3a	50(58.1)	49(92.5)	0(0)	
T3b	17(19.8)	0(0)	18(54.5)	0.000
T3c	10(11.6)	0(0)	10(30.3)	
T4	9(10.5)	4(7.5)	5(15.2)	
N stage (%)				
N0 or Nx	70(81.4)	45(84.9)	25(75.8)	0.289
N1	16(18.6)	8(15.1)	8(24.2)	
M stage (%)				
M0	75(87.2)	50(94.3)	25(75.8)	0.029
M1	11(12.8)	3(5.7)	8(24.2)	
Perinephric fat invasion (%)				
Yes	31(36.0)	11(20.8)	20(60.6)	0.000
No	55(64.0)	42(79.2)	13(39.4)	
Renal pelvis invasion (%)				
Yes	10(11.6)	5(9.4)	5(15.2)	0.647
No	76(88.4)	48(90.6)	28(84.8)	
Thrombus level (%)				
Renal vein	53(61.6)	53(100)	0(0)	
I	16(18.6)	0(0)	16(48.5)	0.000
II	11(12.8)	0(0)	11(33.3)	
III	6(7.0)	0(0)	6(18.2)	
Invasion of IVC wall (%)				
Yes	12(14.0)	0(0)	12(36.4)	0.000
No	74(86.0)	53(100)	21(63.6)	
Neoadjuvant-molecular-targeted therapy (%)				
Yes	2(2.3)	0(0)	2(6.1)	0.144
No	84(97.7)	53(100)	31(93.9)	

All patients in this cohort received radical nephrectomy and tumor thrombectomy. The mean duration of surgery was 3.6 ± 1.1 h (range 1.4–6.1), and 51 patients received blood transfusion with a mean 4.0 ± 2.3 (range 1–15) units. No patient died intraoperatively. The complication rate was 36.0%, but major complications occurred only in 13 (15.1%) patients, including pulmonary embolism in one, acute renal insufficiency in six, ileus in two, re-exploration due to acute postoperative hemorrhage in two, acute myocardial infarction in one, and heart failure in one. An 80-year-old patient with left renal cell carcinoma and level III tumor thrombus underwent extracorporeal circulation. Due to the presence of preoperative renal insufficiency and anemia in this aged patient, we opted not to clamp the right renal vein intraoperatively with the assistance of extracorporeal circulation. There was no decline in renal function after radical nephrectomy and tumor thrombectomy in this patient. After a 32-month recurrence-free survival, right adrenal metastasis was observed. The patient then received tyrosine kinase inhibitors as treatment; thus far, the overall survival time for this patient has been 46 months.

**Figure 1:**
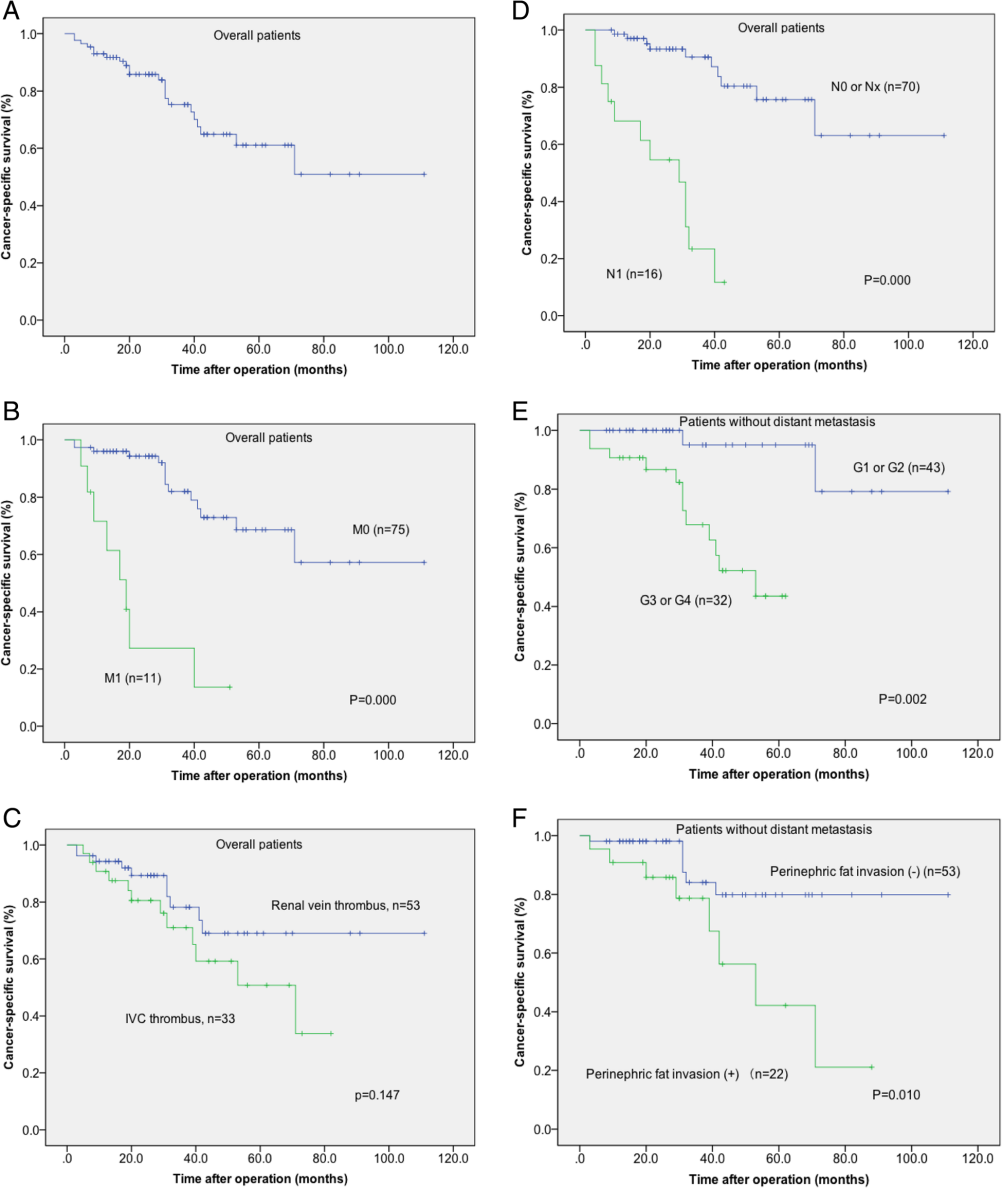
**Kaplan-Meier cancer-specific survival in patients with RCC and venous tumor thrombus. (A)** Overall patients; **(B)** presence of metastasis in overall patients (M0 versus M1); **(C)** the thrombus level in overall patients (renal vein versus IVC); **(D)** presence of lymph node invasion in overall patients (N0 or Nx versus N1); **(E)** tumor grade in patients without distant metastasis (G1–G2 versus G3–G4); **(F)** presence of perinephric fat invasion in patients without distant metastasis (invasion versus non-invasion).

A total of 71 (82.6%) patients received cytokine therapy postoperatively, usually with interferon-α 3 million IU 3 days per week for 3 months or combining it with low-dose interleukin-2. The most common site of metastasis was the lung, followed by bones and liver. A total of 12 patients who were observed to have local recurrence or distant metastasis postoperatively received tyrosine kinase inhibitors (including sunitinib and sorafenib). The 1-, 3-, and 5-year CSS rates for all patients were 93.0%, 70.9%, and 58.1%, respectively (Figure 1A). The 1-, 3-, and 5-year CSS rates for patients without distant metastasis at presentation were 95.3%, 82.6%, and 68.6%, respectively. We found that the CSS rates in patients without distant metastasis at presentation were significantly higher than those in patients with metastasis (Figure 1B). The median survival of all patients and patients without distant metastasis at presentation were undefined. For all patients, there was no significant difference in CSS between the renal vein thrombus group and the IVC thrombus group (Figure 1C); however, there was a significant difference in CSS between the patients with N1 and N0/x (Figure 1D). Corresponding Kaplan-Meier curves for CSS in patients without distant metastasis depending on the presence of perinephric fat invasion and tumor grade are shown in Figure 1E,F. Considering the above two factors, there were statistically significant differences in CSS.

In terms of several prognostic factors, the following were significantly associated with CSS in all patients on univariate analysis: clinical symptoms, ECOG-PS, tumor grade, T stage, lymph node invasion, distant metastasis at presentation, perinephric fat invasion, and invasion of IVC wall. Among these significant prognostic factors, multivariate Cox regression analysis showed that lymph node invasion, distant metastasis at presentation, and invasion of IVC wall were the independent prognostic factors for CSS in all patients (Table 2). The data of patients without distant metastasis at presentation were analyzed separately; univariate analysis showed that ECOG-PS, tumor grade, T stage, lymph node invasion, perinephric fat invasion, and invasion of IVC wall were statistically significant predictors for CSS. Multivariate Cox regression analysis still showed that tumor grade, lymph node invasion, and perinephric fat invasion were the independent factors for predicting CSS in patients without distant metastasis at presentation (Table 2).

**Table 2:** **Univariate and multivariate Cox proportional hazard analyses for cancer-specific survival**

	**All patients**				**Patients without distant metastasis**			
	**Univariate**		**Multivariate**		**Univariate**		**Multivariate**	
	**HR (95% CI)**	***P*** **value**	**HR (95% CI)**	***P*** **value**	**HR (95% CI)**	***P*** **value**	**HR (95% CI)**	***P*** **value**
Age, years (<60 vs ≥60)	0.69(0.31–1.51)	0.35			0.53(0.19–1.48)	0.23		
Gender (male vs female)	0.58(0.26–1.29)	0.18			0.42(0.15–1.17)	0.098		
Laterality (left vs right)	1.43(0.64–3.18)	0.38			1.29(0.45–3.70)	0.63		
Clinical symptom(yes vs no)	3.14(1.25–7.90)	0.018	1.05(0.35–3.11)	0.93	1.72(0.61–4.86)	0.31		
Tumor size, cm (<7 vs ≥7)	0.60(0.25–1.46)	0.26			0.64(0.22–1.90)	0.42		
ECOG-PS (2–3 vs 0–1)	6.32(2.86–13.9)	0.000	1.22(0.30–4.98)	0.78	4.90(1.73–13.9)	0.003	2.43(0.89–8.67)	0.062
Histological type (clear vs others)	0.71(0.44–2.15)	0.24			0.72(0.24–2.11)	0.55		
Tumor grade (G3–G4 vs G1–G2)	5.41(2.01–14.6)	0.001	2.44(0.74–8.05)	0.14	4.15(1.30–13.3)	0.016	4.18(1.43–12.9)	0.010
T stage (T3c–T4 vs T3a–T3b)	7.29(3.17–16.8)	0.000	4.17(0.85–20.3)	0.78	6.05(1.96–18.7)	0.002	3.74(0.77–11.2)	0.612
N stage (N1 vs N0 or Nx)	13.9(5.45–35.7)	0.000	5.44(1.50–19.7)	0.010	11.0(3.60–33.9)	0.000	6.74(1.39–32.7)	0.018
M stage (M1 vs M0)	16.0(6.34–40.4)	0.000	5.12(1.29–20.3)	0.020	-	-		
Perinephric fat invasion (yes vs no)	3.99(1.76–9.05)	0.001	1.48(0.49–4.44)	0.48	3.01(1.09–8.31)	0.034	4.92(1.29–18.7)	0.019
Renal pelvis invasion (yes vs no)	1.15(0.42–3.10)	0.79			2.02(0.71–5.75)	0.19		
Thrombus level (I–III vs 0)	1.68(0.76–3.68)	0.197			1.09(0.39–3.08)	0.87		
Invasion of IVC wall (yes vs no)	6.16(2.62–14.5)	0.000	4.81(1.02–12.1)	0.048	5.10(1.39–18.8)	0.014	1.61(0.59–16.1)	0.073
Neoadjuvant-molecular-targeted therapy (yes vs no)	0.27(0.0–251.3)	0.52			-	-		

## Discussion

The only possible cure for renal cell carcinoma complicated by tumor thrombus is surgery. With advances in surgical techniques and instruments, the surgical indications for RCC complicated by tumor thrombus have been widened and, meanwhile, surgical safety has been improved. The use of laparoscopic and robot-assisted radical nephrectomy and thrombectomy has also been reported [14]. Nevertheless, because of the relatively high incidence of perioperative complications and mortality, the surgical treatment of RCC complicated by tumor thrombus remains challenging. Proactive surgical treatments may improve the prognosis and survival of patients. Adam et al. analyzed 390 patients with tumor thrombus who did not undergo surgical treatment and found their median and 1-year disease-specific survival to be only 5 months and 29%, respectively, which were significantly poorer outcomes than the figures of those who underwent surgical treatment [15]. Another study showed the median survival to be 60 months in patients with RCC and VTT who underwent surgery and 8.2 months in those who did not undergo surgery, and the 5-year overall survival rates to be 54% and 0%, respectively [16]. However, it is still controversial as to whether patients with metastasis should be treated surgically. It was previously recommended that patients with IVC thrombus and distant metastasis should not undergo highly traumatic surgery, because these patients will not survive for long periods [17]. On the other hand, some other studies have shown that, while surgery may not achieve radical cure in patients with metastasis, it usually helped relieve clinical symptoms and improve the quality of life in patients. Moreover, cytoreductive surgery plus interferon can prolong the survival time of patients [18,19]. With the advent of molecular-targeted therapy, the combination of surgical treatment and targeted therapy may improve the long-term survival of patients. Therefore, we believe that proactive surgical treatment is a rational choice for patients with RCC and VTT if their physical status permits.

In the present study, the 1-, 3-, and 5-year CSS rates were 93.0%, 70.9%, and 58.1%, respectively, in all these patients, and were 95.3%, 82.6%, and 68.6%, respectively, in those without metastasis on diagnosis of the cancer. These figures were better than those reported previously [3,9,20]. Such differences may arise from the fact that more patients with renal venous tumor thrombus only were included in our study, and the proportion of patients who underwent surgery within the past 3 years was relatively high in the present study. With improvements in surgical techniques and perioperative management, as well as the advent of targeted drugs, the overall survival of patients with RCC and VTT has increased in recent years. Additionally, 71 of the patients included in this study (82.6%) received cytokine therapy. Whether cytokine therapy played a role, or whether Chinese patients responded to cytokines better than did non-Chinese people, is yet to be investigated. Lymph node invasion, distant metastasis, and IVC wall invasion were suggested as independent predictors for CSS and overall survival (OS) [3,16,21,22], and these factors predicted a poor prognosis. This is basically in agreement with the results of the present study (Table 2). Meanwhile, the present study showed that, in the presence or absence of metastasis on diagnosis of the cancer, lymph node invasion correlated significantly with a poor prognosis. In the case of caval vein wall invasion found intraoperatively, complete resection of the invaded caval vein wall prolonged the survival time significantly; if necessary, the IVC wall can be repaired using a graft to prevent IVC stenosis [23]. For non-metastasis patients, multivariate analysis indicated the tumor grade and perinephric fat invasion to be an independent predictor of a poor prognosis in Chinese patients with RCC and VTT. Obviously, a higher pathological grade usually suggests a more malignant and progressive tumor. Some other studies also showed a poorer prognosis in patients with G3 or G4 tumors than in those with G1 or G2 tumors [22,24]. More frequent postoperative reexamination and follow-up should be considered for patients with tumors of higher grades. Perinephric fat invasion usually suggests a greater invasiveness of tumor. Although some studies have not shown perinephric fat invasion to be an independent prognostic predictor [9,22], two multicenter, large-scale studies have demonstrated that perinephric fat invasion correlated significantly with a poor prognosis and was an independent prognostic predictor for CSS in all patients [6,25]. The present study suggested perinephric fat invasion to be an independent prognostic predictor in Chinese patients with RCC and VTT but without metastasis; however, perinephric fat invasion was a statistically significant predictor for CSS of all patients in univariate but not in multivariate analysis (Table 2).

Whether the level of VTT is a prognosis predictor remains controversial. The present study did not show significant differences in CSS between the renal vein group and the IVC group. Some other studies have made similar findings to ours, revealing no significant correlation between the level of tumor thrombus and prognosis [3,26,27]. Moreover, according to the 2002 TNM classification, tumor thrombus invasion of the renal vein and IVC is classified as T3b. On the contrary, many studies have shown that the prognosis was significantly better in patients with tumor thrombus invasion of the renal vein alone than in those with tumor thrombus invasion of the IVC [28,29]. Therefore, the classification of tumor thrombus invasion of the renal vein has been changed from T3b to T3a according to the 2009 TNM classification. Wagner et al. analyzed the data of 1,192 patients with venous tumor thrombus from 13 European centers retrospectively and found that the overall survival time was significantly longer in patients with tumor thrombus invasion of the renal vein alone than in those with tumor thrombus invasion of the IVC, but the level of tumor thrombus in the IVC did not significantly predict long-term OS [30]. The patients included in this study did not have level IV tumor thrombus. Moreover, due to economic considerations, 9 of the 12 patients with postoperative relapse who received targeted therapy were preoperatively complicated with IVC tumor thrombus. These may partly explain why CSS was not significantly poorer in the IVC group than the renal vein group in our study. Nevertheless, the surgical risk and the incidence of perioperative complications were significantly higher in patients with tumor thrombus invasion of the IVC than in those with tumor thrombus invasion of renal vein only.

The pre- and post-operative use of molecular-targeted therapy regimens remains controversial in patients with RCC and VTT. In our study, two patients received preoperative neoadjuvant therapy and lowered level IV tumor thrombus to level III and, subsequently, underwent radical nephrectomy and tumor thrombectomy. On the other hand, in some patients, targeted therapy did not lead to changes in tumor thrombus and metastatic lesions, and the condition progressed and surgical treatment became impossible. These patients were not included in the present study. To date, studies on molecular-targeted therapy have been carried out mainly in small numbers of patients with RCC and VTT. Nicholas et al. reported 25 patients with RCC and IVC tumor thrombus who received molecular-targeted drugs. Eleven patients (44%) were found with tumor thrombus length shrinkage and only three patients (12%) with lowering of the thrombus level. Among the patients with tumor thrombus length shrinkage, only one case (4%) was potentially affected to change the surgical approach. On the other hand, seven patients suffered from tumor thrombus prolongation [31]. Therefore, for patients with RCC and VTT, neoadjuvant-targeted molecular therapies should be selected with caution. Meanwhile, more prospective studies, and more reliable molecular markers, are required to be discovered to help decide who will benefit from molecular-targeted therapy.

The present study bears some limitations. First of all, this is a single-center, retrospective study based on a small-sample size. Secondly, the surgical cases spanned a decade, and different surgeons were involved, which resulted in heterogeneity of the case data and treatments. Besides surgery, molecular-targeted therapy may significantly affect the survival of patients with RCC and VTT. All these may lead to statistical biases. Nevertheless, the present study is a long-term follow-up study with the largest sample size of Chinese patients with RCC and VTT. By undertaking more Asian multicenter studies, we hope to improve the therapeutic efficacy of the treatment of renal cell carcinoma in Asian patients.

## Conclusions

The present study showed that the tumor prognosis was similar in Chinese patients with RCC and VTT to patients in other countries. Proactive surgical treatment may relieve clinical symptoms and benefit patient survival. Consistent with other studies, the present study showed distant metastasis, lymph node invasion, and IVC wall invasion to be independent prognostic factors. In addition, for Chinese non-metastatic patients, perinephric fat invasion and pathological grade correlated significantly with tumor prognosis. The advent of the era of molecular-targeted therapy brings a brighter future for patients with RCC and VTT. More multicenter, prospective studies are needed to evaluate the therapeutic efficacy of these therapies.

## Abbreviations

RCC: Renal cell carcinoma
VTT: Venous tumor thrombus
CSS: Cancer-specific survival
IVC: Inferior vena cava
PS: Performance status
TNM: Tumor-node-metastasis
ISUP: International Society of Urological Pathology
CT: Computed tomography
MRI: Magnetic resonance imaging
HR: Hazard ratio
ECOG: Eastern Cooperative Oncology Group
OS: Overall survival.
